# Analysis of conservative tracer measurement results inside a planted horizontal subsurface flow constructed wetland filled with coarse gravel using Frechet distribution

**DOI:** 10.1007/s11356-020-10246-9

**Published:** 2020-09-22

**Authors:** Ernő Dittrich, Mihály Klincsik, Dávid Somfai, Anita Dolgos-Kovács, Tibor Kiss, Anett Szekeres

**Affiliations:** 1 Faculty of Engineering and Informatics, Department of Environmental Engineering, University of Pécs, Boszorkány u. 2, Pécs, H-7624 Hungary; 2 Faculty of Engineering and Informatics, Department of Mathematical Sciences, University of Pécs, Boszorkány u. 2, Pécs, H-7624 Hungary; 3 Hidro-Consulting Ltd., Budai Nagy Antal u. 1, Pécs, H-7624 Hungary

**Keywords:** Frechet distribution, Inverse Gaussian distribution, Subsurface flow constructed wetlands, Transport processes, Tracer test, Internal hydraulic variability

## Abstract

We worked out a method in Maple environment to help understand the difficult transport processes in horizontal subsurface flow constructed wetlands filled with coarse gravel (HSFCW-C). With this process, the measured tracer results of the inner points of a HSFCW-C can be fitted more accurately than with the conventionally used distribution functions (Gaussian, Lognormal, Fick (Inverse Gaussian) and Gamma). This research outcome only applies for planted HSFCW-Cs. The outcome of the analysis shows that conventional solutions completely stirred series tank reactor (CSTR) model and convection-dispersion transport (CDT) model do not describe the internal transport processes with sufficient accuracy. This study may help us develop better process descriptions of very complex transport processes in HSFCW-Cs. Our results also revealed that the tracer response curves of planted HSFCW-C conservative inner points can be fitted well with Frechet distribution only if the response curve has one peak.

## Introduction

Constructed wetlands (CWs)—also known as treatment wetlands—are engineered systems for wastewater treatment. Constructed wetlands have a very low or zero energy demand; therefore, operation and maintenance costs are significantly reduced compared with conventional treatment systems (Almuktar et al. 2018).

There are two main types of constructed wetlands: free-surface flow systems (FSF-CW) and subsurface flow systems (SSF-CW). SSF-CWs can be further divided according to the direction of the wastewater flow. Wastewater in SSF-CWs runs either horizontally (in HSSF-CWs) or vertically (in VSSF-CWs) towards the filter media. In VSFCWs, there is unsaturated, non-permanent flow, and in HFSFCWs there is saturated non-permanent flow (Wu et al. 2015; Valipour and Ahn 2016). In our experiments and calculations, only HFSFCWs were considered. We investigated HFSFCWs using coarse gravel as filter media (HFSCW-C). Constructed wetlands can treat a wide variety of polluted water, including municipal, domestic, agricultural or industrial wastewaters (Vymazal 2009).

There are important differences between the ideal and the actual flow. One of the reasons is weather conditions, such as rainfall (Kadlec 1997; Kadlec 1999; Rash and Liehr 1999), evapotranspiration (Galvão et al. 2010; Beebe et al. 2014) and snow melting can have a huge impact on the flow within constructed wetlands. Another important factor is the construction of the CW: the differences in porosity and hydraulic conductivity of filter media in volume and over time (Dittrich and Klincsik 2015a; Licciardello et al. 2019), the active volume of the porous system (Goebes and Younger 2004) and the inlet and outlet positions (Alcocer et al. 2012; Wang et al. 2014; Okhravi et al. 2017). The last is the clogging processes, which are caused by solids accumulation (Carballeira et al. 2016; Lancheros et al. 2017; Liu et al. 2019), biofilm development (Button et al. 2015; Aiello et al. 2016; Vymazal 2018; de Matos et al. 2018), and root density and distribution (de Paoli and von Sperling 2013; Tang et al. 2017).

Due to the factors mentioned above, the hydrodynamic modelling of SFCWs is a challenging task for experts. In these constructions, biofilm activity and root density can be very intensive, and more importantly, the biofilm development and root system growth over time may also be significantly more rapid (Samsó and Garcia 2013; Rajabzadeh et al. 2015). These processes can affect the microporous system, hydraulic conductivity and clogging processes as well (Tanner and Sukias 1995). It is quite challenging and often problematic to estimate these processes or even further, to incorporate these factors into a model.

Conservative tracer tests are commonly used to analyse the hydraulic behaviour of constructed wetlands (Levenspiel 1972). Scientists have frequently analysed SFCWs with conservative tracer tests used as experimental tools to gain more detailed information about the internal hydrodynamics of constructed wetlands (Netter 1994; Suliman et al. 2006; Barbagallo et al. 2011; Wang et al. 2014). Our method was also based on tracer tests. Conservative tracer tests allow for calculations of the hydraulic retention time (HRT) and dispersion coefficient (*D*) of a hydraulic system. Some scientists have also conducted the same tests in HSFCWs with the same goal.

Netter (1994) measured two horizontal subsurface flow constructed wetlands. He conducted tracer tests on each CW. They were filled with different, homogeneously mixed media, gravelly sand and sandy gravel, and both filter materials contained fractions of clay and silt. Samples were taken from inside the CWs and at the effluent point as well. The conclusion was that the hydraulic performance varied considerably inside the system due to the detrimental length to width ratio. Initially, there was plug flow with little longitudinal dispersion in this CW.

Breen and Chick (1995) completed a more itemised tracer test as they measured tracer concentration values at the bottom and at the top section of the filter media. Similar hydraulic behaviour was observed as described by Netter (1994); however, the authors attributed it to dead zones and hydraulic shortcuts.

Liu et al. (2018) investigated the effect of solids accumulation and root growth on the hydrodynamics of HSFCWs. They used three laboratory-scale HSFCWs. The tracer was fluorescein sodium. Samples were taken from two points and three different substrate depths. The results indicated that the presence of plant root restricted the water flow in the top layer, leading to the preferred, bottom-flow phenomenon.

Birkigt et al. (2017) investigated the flow and transport processes on a pilot-scale, horizontal subsurface constructed wetland with tracer tests (bromide, deuterium oxide and uranine). There was one sampling point inside the CW; samples were obtained from three depths. The results showed that the preferred flow distribution consisted of 65–70% of mass flowing along the bottom, and 14–18% and 16–17% of mass at the middle and top levels.

The most commonly used SFCW modelling programs have been HYDRUS2D and FITOVERT (Wang et al. 2011; Kumar and Zhao 2011); nevertheless, these softwares also need further development.

Batchelor and Loots (1996) tried to fit completely stirred series tank reactor (CSTR) and convection-dispersion transport (CDT) models too to their tracer test results which yielded bad fitting results; the reason of which the authors did not exactly know. Chazarenc et al. (2003) investigated with fitting CSTR and CDT models as well, which fortunately, resulted in good fitting with CSTR models 9 out of 10 times. Nonetheless, the important parameters, for example, porosity and hydraulic conductivity, were estimated values only. King et al. (1997) conducted a conservative tracer analysis of a gravel-filled HSFCW. They fitted CSTR and CDT models as well; they found bad fittings too. Hydrus 2D uses CSTR and CDT models also at the transport module of the software (Langergraber and Simunek 2011; Langergraber et al. 2009; Toscano et al. 2009); results published nonetheless indicate that the module needs further development.

Several international researchers have shown that CDT and CSTR models do not correlate precisely with tracer test results in HFSCWs (Batchelor and Loots 1996; King et al. 1997; Kumar and Zhao 2011). The CDT model uses Inverse Gaussian distribution, and the CSTR model uses Gamma distribution. Taking into consideration the irregularities in previous studies, we tried to find closer correlations among other distribution function types.

## Materials and methods

The tracer measurements were made at a HSFCW-C in Hódmezővásárhely, Hungary. Scientists used different tracers, in two cases NaBr (Netter 1994; Tanner and Suikas 1995), in one of the cases tritium (Netter 1994), in another case a special fluorescent substance (eriochrome acid red) (Breen and Chick 1995) and in four cases LiCl (Schierup et al. 1990; Netter 1994; King et al. 1997; Rash and Liehr 1999).

We chose LiCl as a conservative tracer. The absorption capacity of the filter media for LiCl was tested in the Environmental Technological Laboratory of the University of Pécs. The findings indicated that LiCl is applicable as a conservative tracer in the examined construction. More details of the treatment plant and the tracer test can be found in Dittrich and Klincsik (2015a).

**Fig. 1:**
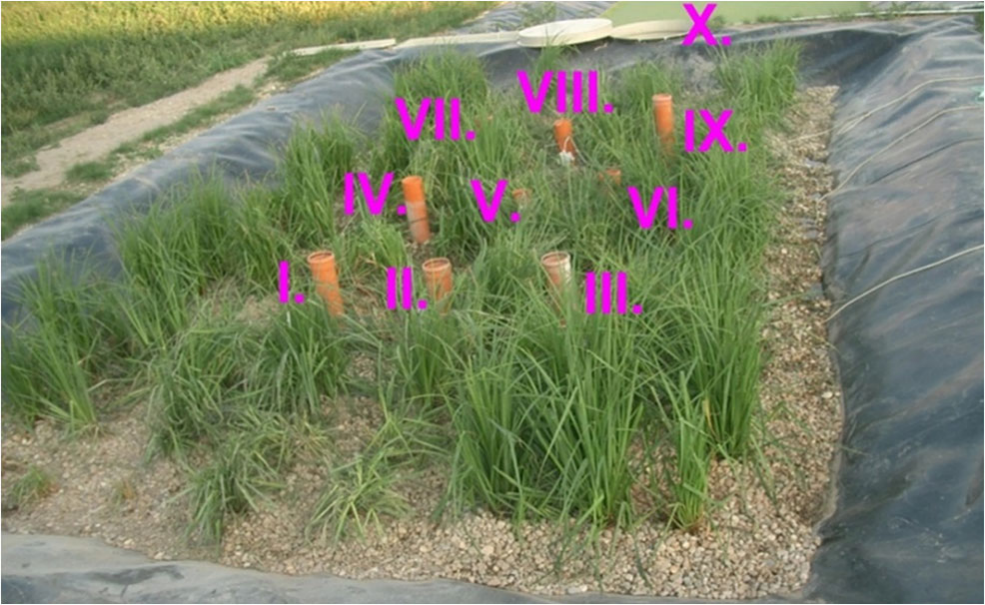
Measurement points in the HSFCW-C in Hódmezővásárhely, Hungary

Inside the CW, there were 9 sample points, and samples were collected at the effluent. These points are demonstrated in Fig. 1. The LiCl concentration values of the samplings were measured with a UNICAM Solaar M atomic absorption device.

The results gained at the effluent point have already been published (Dittrich and Klincsik 2015a).

The measured concentration-time value pairs and other relevant measurements are summarised in Appendix 1. We have made four separate measurements at different times and in different seasons. The measurements received S/1, S/2, S/3 and S/4 reference numbers for easier documentation. The main data of our own tracer measurements are summarised in Appendix 1.

We found five applicable distribution function types (Fatigue Life, Lognormal, Frechet, Pearson5 and Inverse Gaussian); for detailed analysis, we used EasyFit program. More information on the selection criteria for the functions can be found in Dittrich and Klincsik (2015a). Subsequently, a more accurate and specific fitting method was established in Maple environment to ensure accurate comparison of results for these functions. This mathematical method is able to fit the functions to the measurement values with specifically defined conditions. Further details are found in Dittrich and Klincsik (2015a). The mathematical procedure was published in Dittrich and Klincsik (2015a).

Dittrich and Klincsik (2015a) demonstrated that the Frechet distribution is the best-fitting function to effluent point measurement results. The results show that Frechet had the best average *R*^2^ of the effluent measurement point. Only the Pearson5 *R*^2^ value was sufficiently good, nevertheless, lower than Frechet values The present article aims to investigate which is the best-fitting distribution type in inner points.

In tracer test analysis, scientists do not usually measure porosity; instead, they use the porosity value of newly built filter media before starting the operation or they estimate porosity (Schierup et al. 1990; Tanner and Sukias 1995). In our study (Dittrich and Klincsik 2015a), by measuring the porosity of the analysed HSFCW-C, a very precise analysis was performed. Our results show that the effective porosity of the HSFCW-C decreased by more than 50% in the first 6 months as a result of intense biological activity and root growth. These data were used for the analysis of the transport processes. Detailed information about these results can be found in Dittrich and Klincsik (2015a).

## Results and discussion

During the course of our work, the following functions were fitted to the data sets in Appendix 1: Fatigue Life, Frechet, Inverse Gauss, LogNormal, and Pearson5. A customised program in the Maple software was applied for the fittings. The input values of the program are shown in Appendix 1. Tables 6, 7, 8, and 9 show concentration and time values, as well as areas under the predefined function. Appendix 2 contains the *R*^2^ value results at each point (Table 10). Appendix 3 shows all images of the fittings (Tables 11, 12, 13, 14, 15, 16, 17, and 18).

**Fig. 2:**
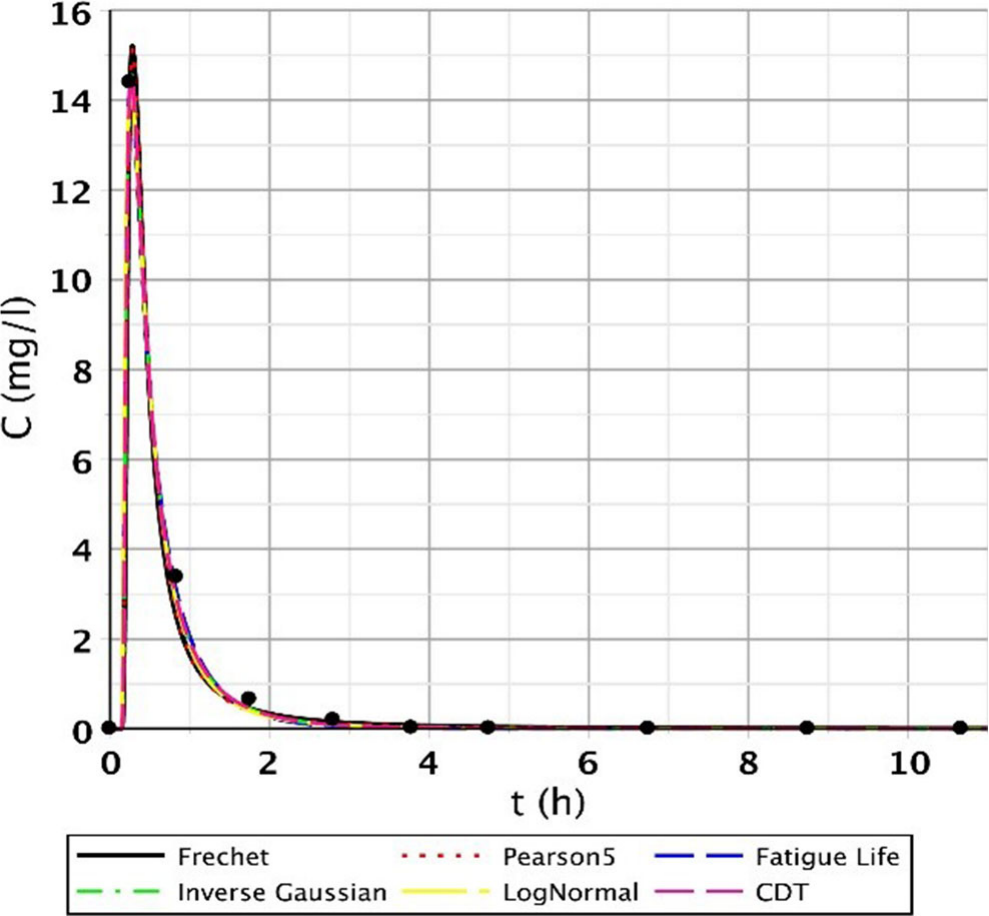
Fitting results of the five distributions and CDT model on S/1 measurement I. top point

**Fig. 3:**
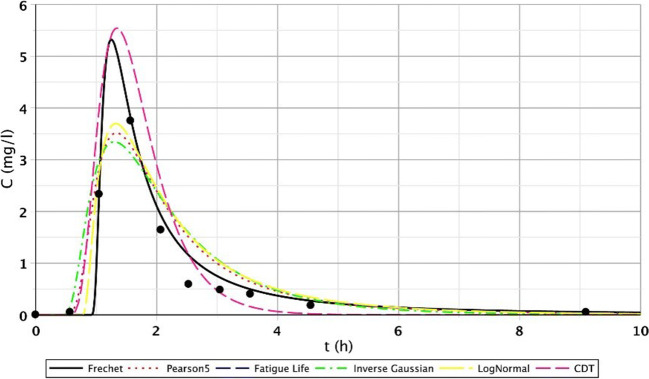
Fitting results of the five distributions and CDT model on S/1 measurement V. bottom point

**Fig. 4:**
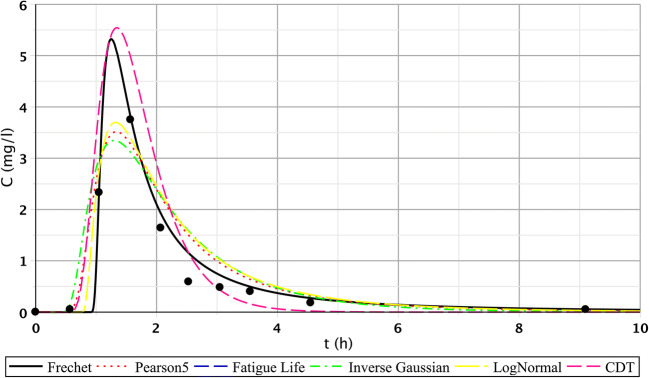
Fitting results of the five distributions and CDT model on S/1 measurement VII. bottom point

The S/1 measurement is interesting as the sampling data refer to the CW with only 2 days of age (the installation took place on September 01, 2007). Generally, for points I.–III., we obtained good results. These points were characterised by fast-rising, peaked curves. The second segment (IV.–VI.) had wider, flatter curves due to leakage rate deceleration and mixing processes. Regarding points VII.–IX., the measurement results were no longer included in the run of the function; they also contained smaller and larger jumps; thus, the fitting results significantly deteriorated. Function pictures (Figs. 2, 3, and 4.) support this assessment.

For lower point, VII. was the first where we received bad results as shown in Table 1. There were measurement results, where the functions could not fit well; only the Frechet distribution gave a value above 0.95.

**Table 1:** *R*^2^ values of each function for VII. bottom point

Function type	*R*^2^ values
Fatigue Life	0.8047
Frechet	0.9651
Inverse Gauss	0.8126
LogNormal	0.8481
Pearson	0.8576

**Fig. 5:**
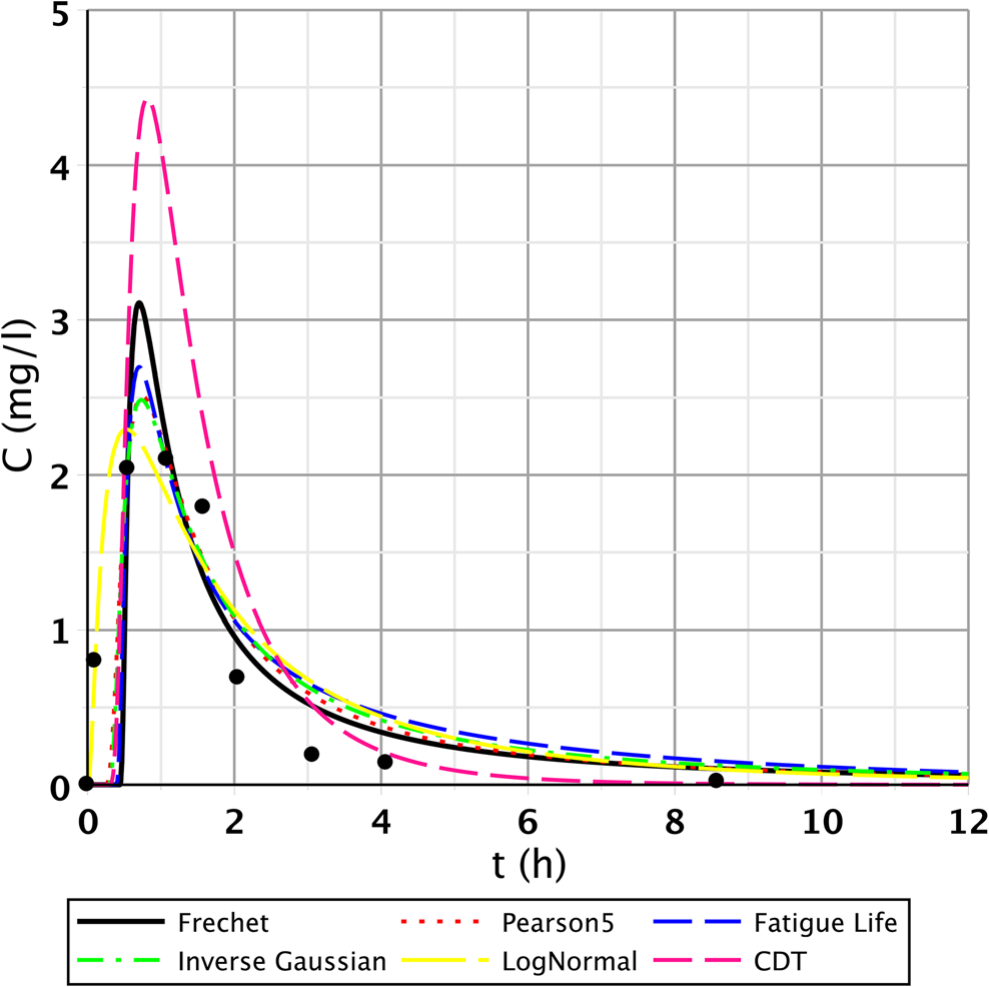
Fitting results of the five distributions and CDT model on S/2 measurement IV. bottom point

**Fig. 6:**
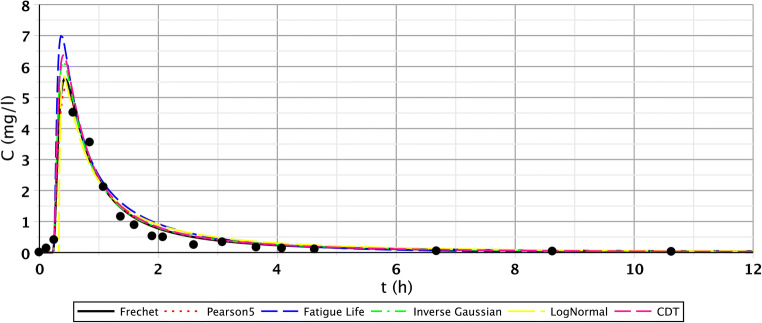
Fitting results of the five distributions and CDT model on S/2 measurement V. bottom point

**Fig. 7:**
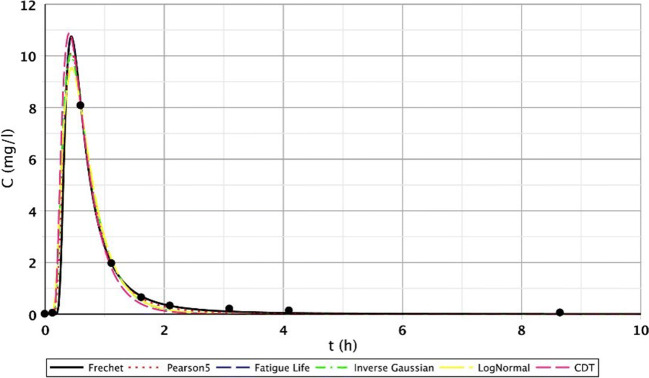
Fitting results of the five distributions and CDT model on S/1 measurement VI. bottom point

The results of the S/2 measurement reflect the evolution of transport processes of the constructed wetland of 1 month age. The first three measuring points produced similarly favourable fittings. However, in the case of the second segment, only point IV. showed flatter functions. For points V.–VI., we got similarly good fittings than at the first section. This observation is probably a consequence of inhomogeneous flow distribution. The inhomogeneous flow distribution means that in this cross section (IV.–VI.), at point IV., the root growth and the biofilm activity caused a slower flow. At points V. and VI., the flow was faster because the roots were less and the biofilm activity was lower. Fitting results of points IV.–VI. are shown in Figs. 5, 6, and 7, which illustrate this statement. Functions of the third section were similar to the results of the first measurement.

Fitting the functions was the most difficult at the measurement of S/3. For the first two sections (in Appendix 3: Table 12, S/3 measurement I.–VI. top point figures and in Table 13 S/3 measurement I. to VI. bottom point figures), the picture of all functions demonstrated that the area under the specified function was too small; only for the third section was it identical to the area drawn by the measurement points. The reason for this observation was that the mechanical pre-treatment of the wastewater did not work well leading to significant clogging in the horizontal flow constructed wetland. Mechanical pre-treatment is a septic tank which helps solids settling. The clogged filter media have been replaced with a new filter media of the same type; thus, the pre-treatment problem was solved, so that the subsequent measurement results would no longer be affected by strong clogging processes.

**Fig. 8:**
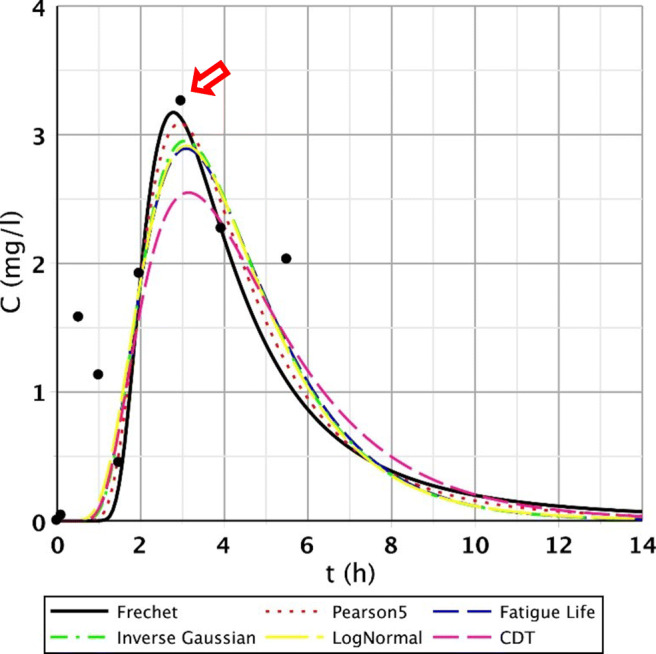
Fitting results of the five distributions and CDT model on S/3 measurement IV. bottom point

**Fig. 9:**
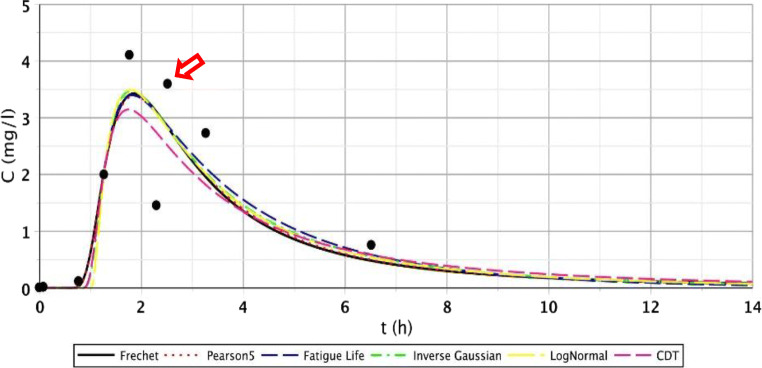
Fitting results of the five distributions and CDT model on S/3 measurement V. top point

**Fig. 10:**
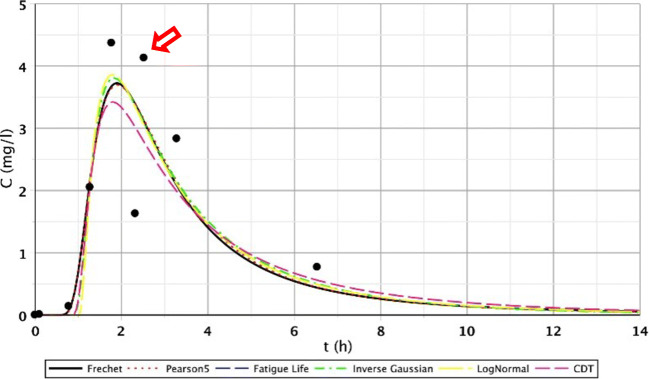
Fitting results of the five distributions and CDT model on S/3 measurement VI. top point

**Fig. 11:**
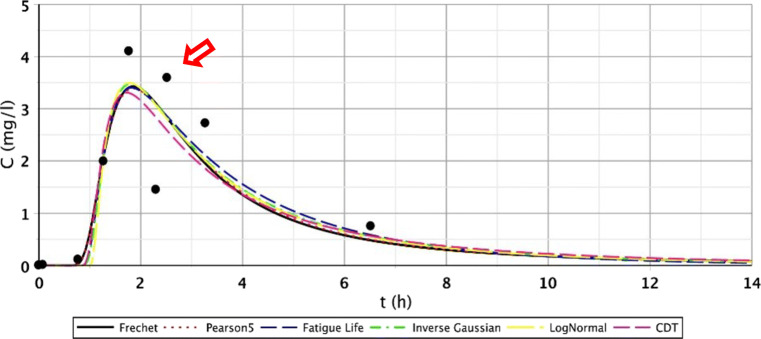
Fitting results of the five distributions and CDT model on S/3 measurement V. top point

The other reason was that the roots of the tufted sedge had sufficiently developed during the first 5 months in the constructed wetland, resulting in further flow distortions. Due to the development of dead zones, intensive biofilm activity and clogging processes, the role of the secondary stream is significant. The presence of the dead zones is mostly indicated by the poor fitting of the Inverse Gaussian function and the elongated tail length of the curve. This is clearly visible on the following figures (Figs. 8, 9, and 10). The red arrows on the following figures (Figs. 8, 9, 10, 11, and 13) show the secondary streams (second peaks).

**Fig. 12:**
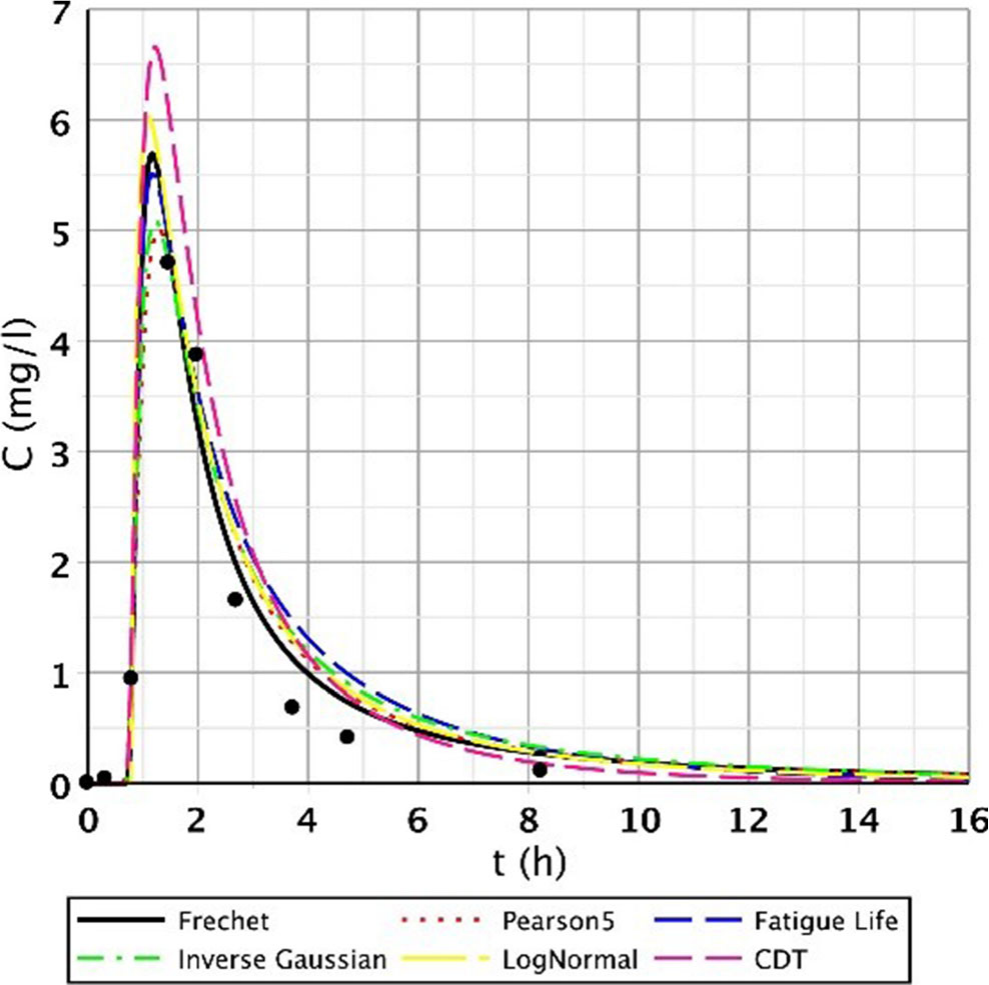
Fitting results of the five distributions and CDT model on S/4 measurement V. top point

**Fig. 13:**
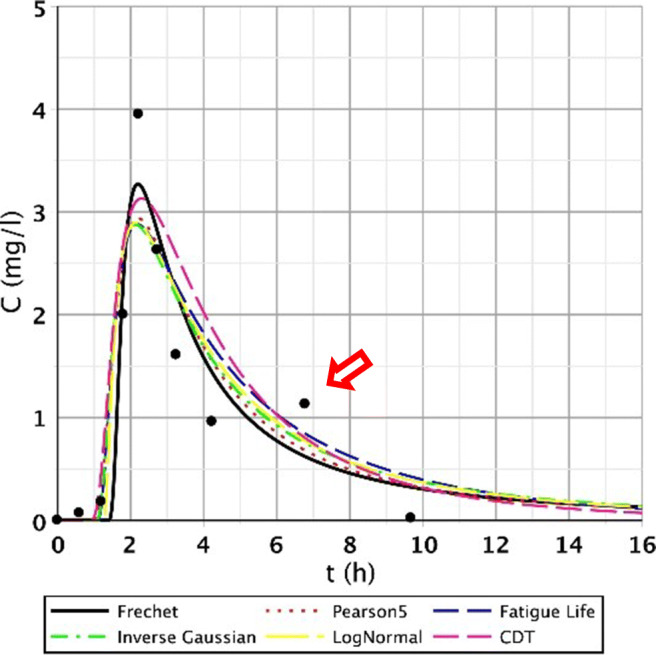
Example of a two-peak function obtained as a result of the measurement of S/4 VIII. bottom point

Figures 8, 9, 10, and 11 clearly demonstrate that if the response curve has two peaks, neither function fits well enough. At these measuring points, the use of the divided convective-dispersive model plays an important role (Dittrich and Klincsik 2015b). The results of the last measurement S/4 (May 29, 2008) provide a better picture due to the result yielded by using new filter media of the constructed wetland. Comparing these measurements with the S/3 measurement results, we can see that clogging has occurred in the CW due to temporary malfunction, resulting in bad fittings (Fig. 11), but as soon as the malfunction stopped, good fittings were achieved (Fig. 12). Compare images of S/3 and S/4 V. fitted results of top point measurements (Fig. 11 and 12):

The results were completely different; nevertheless, we obtained the expected results. After changing the clogged filter media in the constructed wetland to a new one (same type media), the second peak disappeared (in Appendix 3. Table 17, S/4 measurement I.–IX. top points and Table 18, S/4 measurement I.–VII, and IX. bottom points). However, it also became apparent that it originally tried to fit a similar shape function into the measurement points. There is a functional problem for this particular measurement that has to be mentioned. There was a two-peak curve which revealed worse fittings; consequently, the use of the divided convective-dispersive model was necessitated. This model could not only fit the first peak but the second as well, so it had much better fitting results than for example the CDT model (Dittrich and Klincsik 2015b). This point can be seen in Fig. 13.

We investigated the order of functions with the age of the constructed wetland. First, we took the average of each function for each measurement time, as shown in the Table 2. The data in the table refer to the measurements were performed; the duration of each experiment was between 8 and 14 h.

**Table 2:** *R*^2^ averages at individual measurement times for each function in the order of fitting

Distribution type	*R*^2^ values			
	September 02, 2007	October 07, 2007	February 08, 2008	May 29, 2008
Frechet	0.984546	0.973443	0.875733	0.946450
Pearson5	0.967940	0.963841	0.897407	0.913429
LogNormal	0.966497	0.946875	0.898795	0.887550
Fatigue Life	0.961163	0.958451	0.899836	0.879096
Inverse Gauss	0.956540	0.948844	0.902576	0.775451

**Fig. 14:**
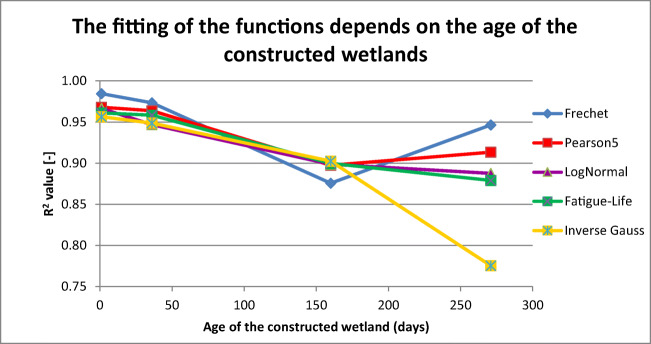
The fitting of the functions depends on the age of the constructed wetland

Table 2 clearly demonstrates that the fitting was adequate for each function in the new constructed wetland, but with the ageing of the wetland, the functions became more and more difficult to follow at the measurement points due to the flow distortions caused by root growth or biofilm activity (highlights indicate results that do not reach 0.95). Appendix 2 shows *R*^2^ results for each point. The degree of the fittings is better than those achieved by using conventional models. We determined this value (0.95) as we thought that above this not only the fitting was good enough but also that this value was higher than the ones used in international studies. Figure 14 shows the evolution of *R*^2^ as a function of the age of the CW. The hypothesis that the degree of inaccuracy increases with the age of the CW is apparent when applying Inverse Gauss, LogNormal and Fatigue Life (Fig. 14, yellow, purple and green lines); for the other functions, it is completely different (Fig. 14, blue and red lines).

It can be stated that the results of the fitting deteriorated with time passing through the distortion of the flow, and the Frechet distribution only gave a good fitting when the measurement curve only had one peak. Further research is required if a response curve has two peaks. For this type of modelling, we have been the first to use the Frechet distribution and Pearson5. To date, researchers have only used the Inverse Gaussian distribution, and we got similar fitting results as other international studies.

**Fig. 15:**
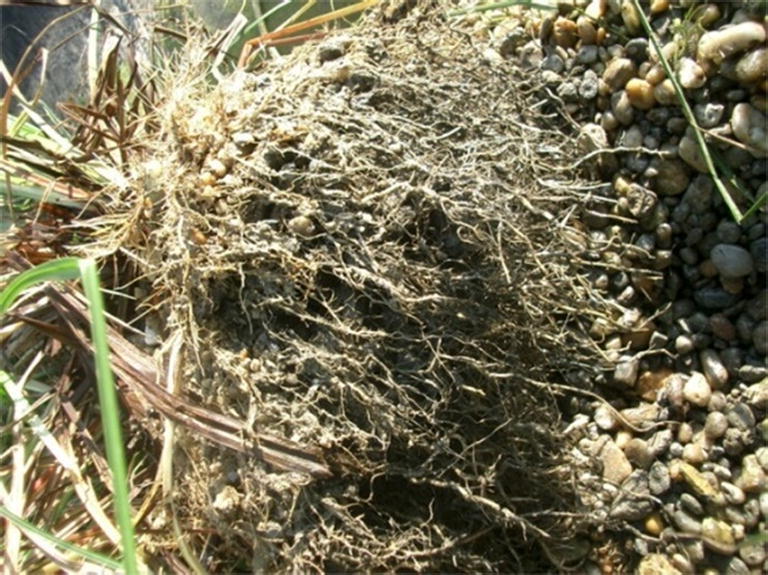
The rhizome systems of sedge form island-like zones in the medium

We investigated the fitting results of the top and bottom measuring points; as we assumed, our results have shown that the values of the top and bottom measurement points may differ according to the position of the unsuitably formed dividing line and the root stratification. First, we measured the length and width of a randomly selected root. The planted Carex Elata has a globular root system (see Fig. 15). Back-mixing zones can form behind these insular root zones causing smaller hydraulic conductivity in such areas; thus, the wastewater needs to change flow direction in the filter media.

Table 3 shows that the bottom points are much more balanced by the fitting of each function. In the case of the top points, however, only Frechet and Pearson5 gave a better fit than in the bottom points; the others were weaker, and when applying the Inverse Gauss, the average *R*^2^ of the top points gave a very bad result. When setting the functions’ fitting order, Frechet and Pearson5 again ranked the first two and Inverse Gauss ranked fifth. In the top layer, with slower flow and denser roots, and consequently, more dead zones and more intense biofilm activity, these factors are difficult to adapt to functions. This means that the main flow is at the bottom. Bonner et al. (2016) and Liu et al. (2018) came to a similar conclusion from their results.

**Table 3:** *R*^2^ average at the lower and upper points

Distribution types	Top		Bottom	
	Average of the *R*^2^	Ranking	Average of the *R*^2^	Ranking
Frechet	0.948576	1	0.941510	1
Pearson	0.936302	2	0.935006	2
Fatigue Life	0.921510	3	0.927763	4
LogNormal	0.917511	4	0.932348	3
Inverse Gauss	0.868013	5	0.923692	5

In Table 4, the second and third columns contain the fitting results and ranking of the effluent point, as published in Dittrich and Klincsik (2015a), while the fourth and fifth columns contain the results and ranking for inner points. It is striking that the order was comparable with the previous measurement results (Table 4, point X.). The two best-fit functions were Frechet and Pearson5; the worst was the Inverse Gauss. The order of LogNormal and Fatigue Life was interchanged; however, when taking a closer look at the results, it appears that the two values are actually very close. The results met our expectations: the results of the inner points’ fittings were very similar to the effluent point fitting results.

**Table 4:** Comparison of the final ranking of internal points with point X. and results from international data

Distribution type	X.	Ranking	I–IX	Ranking
Frechet	0.9867	1	0.9450	1
Pearson5	0.9839	2	0.9357	2
LogNormal	0.9769	*4*	0.9249	*3*
Fatigue Life	0.9779	*3*	0.9246	*4*
Inverse Gauss	0.9714	5	0.8959	5

The results indicate that regarding internal points, the standard deviation of the *R*^2^ average is higher than at the effluent point, and that the internal points gave worse fitting results.

The average difference between the previous results and the internal points was 0.054. It is important to highlight that none of the functions’ average *R*^2^ values reached 0.95; it can be stated, therefore, that none of them fitted perfectly with the measuring points.

## Conclusions

The purpose of our research was to find better-fitted distribution functions than those conventionally used to our conservative tracer test results at the inside points of a Hungarian HSFCW-C. We fitted 5 distribution functions in the Maple software onto tracer test results of our inner points. These 5 function types were chosen from among a large amount of distribution functions (Dittrich and Klincsik 2015a).

We have determined that the Inverse Gauss function ranked 5th in the order of alignment of the functions. In two cases, it was necessary to modify the parameters manually to fit the specified points. The analytical solution of the CDT model is an Inverse Gaussian distribution function. Therefore, it seems clear that the normal CDT model cannot precisely generate a correctly fitting correlation, as the *R*^2^ values did not reach 0.95, and below this value, the fitting did not give the expected results. The error of the CDT model increases with the age of the CW.

The Fatigue Life and LogNormal distributions in the order of alignment will always be 3–4, which means that these two distributions take the third and fourth places at both the effluent and inner points (Table 3 and Table 4). They can be ignored in further investigations, due to bad average *R*^2^ values. The first two places were achieved by Frechet and Pearson5. The averages of *R*^2^ and the fitting images of the functions indicate that the Frechet distribution incorporates the measurement points more eloquently than the Pearson5 distribution. With this process, we proved that the planted HSFCW-C conservative tracer response curves at inner points of CW demonstrate a Frechet distribution. This result is identical to results published about the effluent point of the same CW (Dittrich and Klincsik 2015a). The Frechet distribution proved to be the best fitting only where the measured curve had one peak. Where the measured curve had two peaks, the Frechet distribution did not fit sufficiently well; thus, further research is needed.

Investigating the top and bottom measurement points, we found that the fitting results at the top measuring points revealed much worse fittings than the bottom measuring points. This is possible as the top layer is characterised by slower flows, denser roots, more dead zones and more intense biofilm activity.

We carried out similar measurements in another constructed wetland in Pécs, and aim to publish the results in another article.

One of our main goals with this fitting procedure in Maple environment was to provide a novel, adaptable method of analysis for other types of hydraulic regimes and thereby, to aid scientists in their analysis of transport test results. In our opinion, the presented statistical method can be used for a deeper understanding of several hydrodynamic problems for the solution of which traditional methods have not been successful, mainly hydraulic leakage problems in other media. Our further research direction is to develop a general software that would allow a wider application. One of the main directions of our future research is to find other areas where similar research success could be achieved.

## Figures and Tables

**Table 11:**
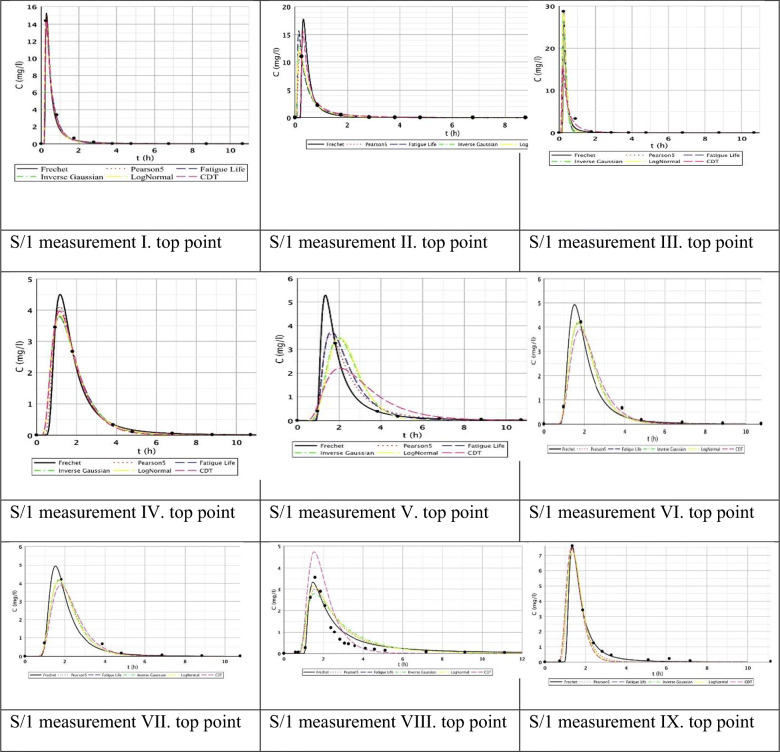
Fitting results of the five distributions and CDT model on S/1 measurement top points

**Table 12:**
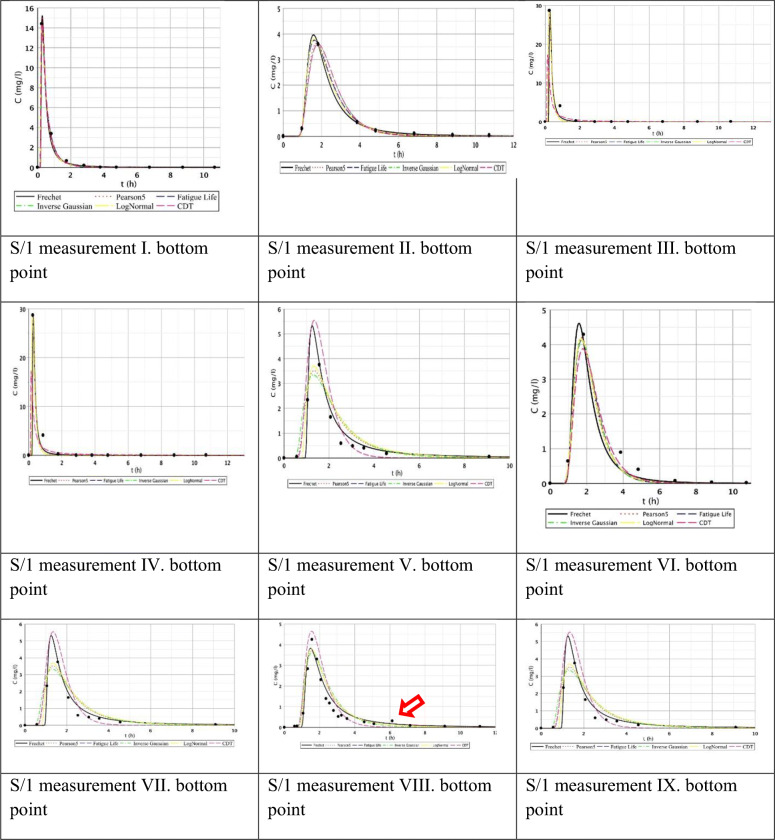
Fitting results of the five distributions and CDT model on S/1 measurement bottom points

**Table 13:**
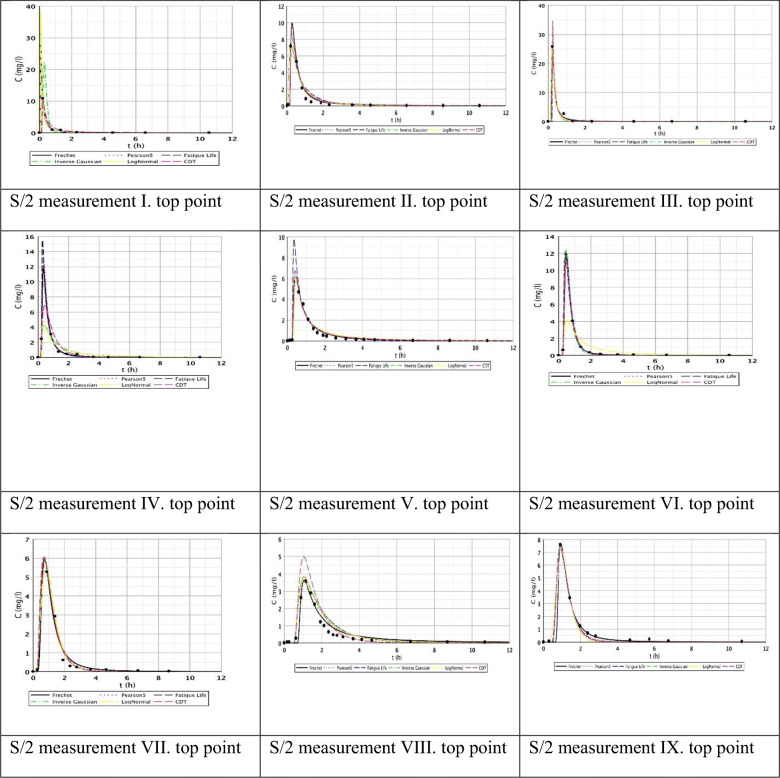
Fitting results of the five distributions and CDT model on S/2 measurement top points

**Table 14:**
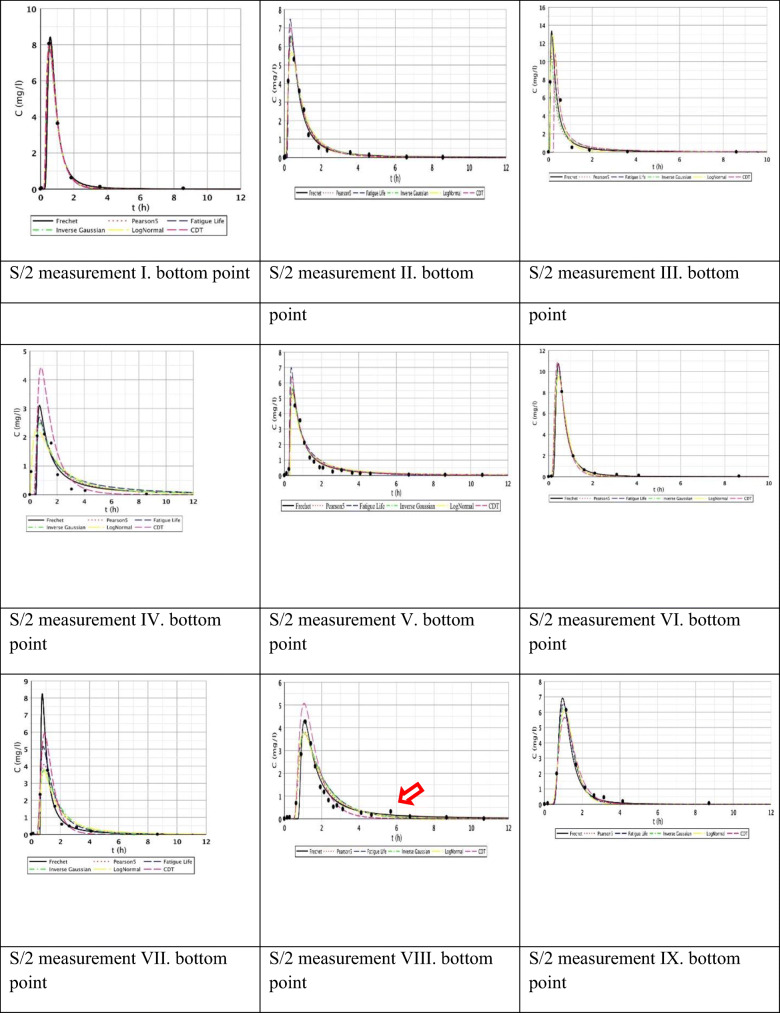
Fitting results of the five distributions and CDT model on S/2 measurement bottom points

**Table 15:**
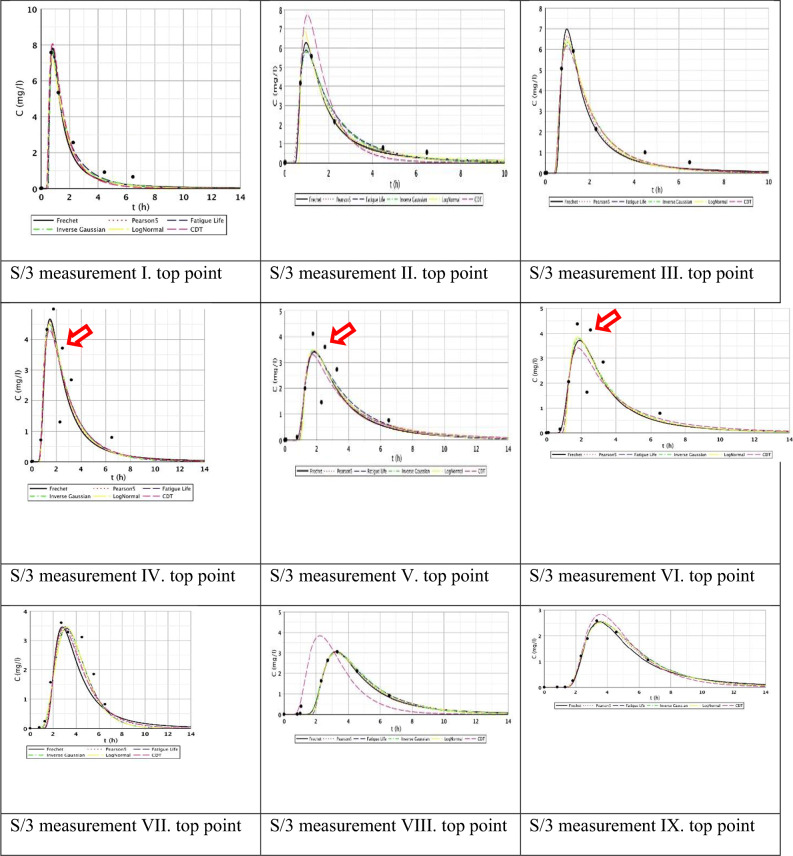
Fitting results of the five distributions and CDT model on S/3 measurement top points

**Table 16:**
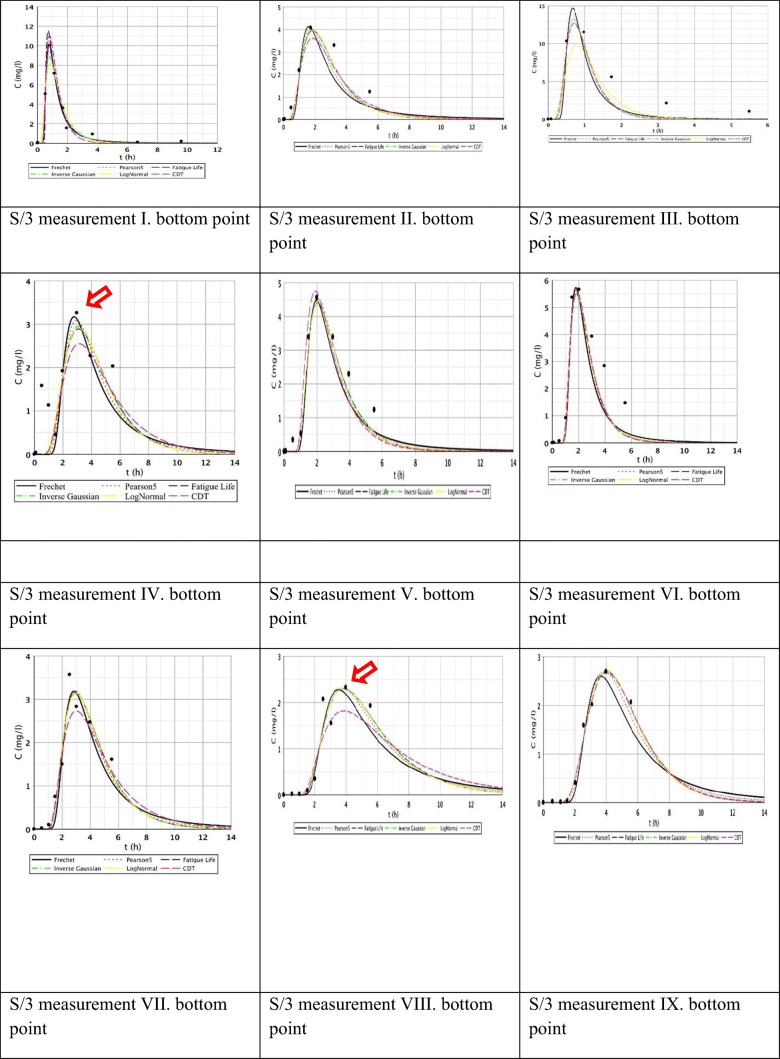
Fitting results of the five distributions and CDT model on S/3 measurement bottom points

**Table 17:**
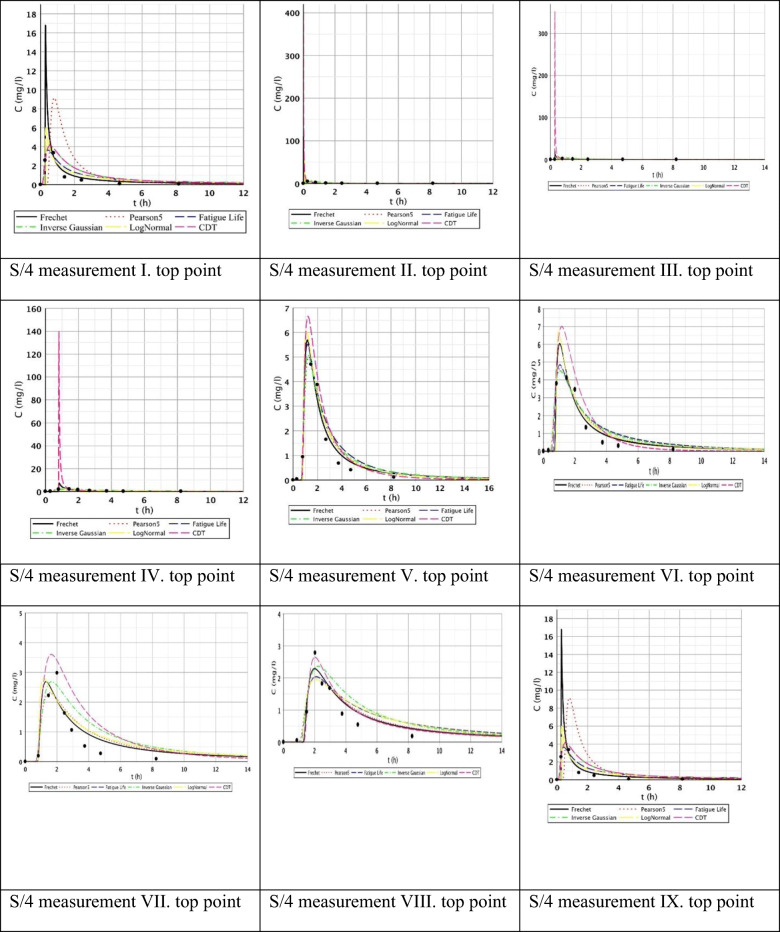
Fitting results of the five distributions and CDT model on S/4 measurement top points

**Table 18:**
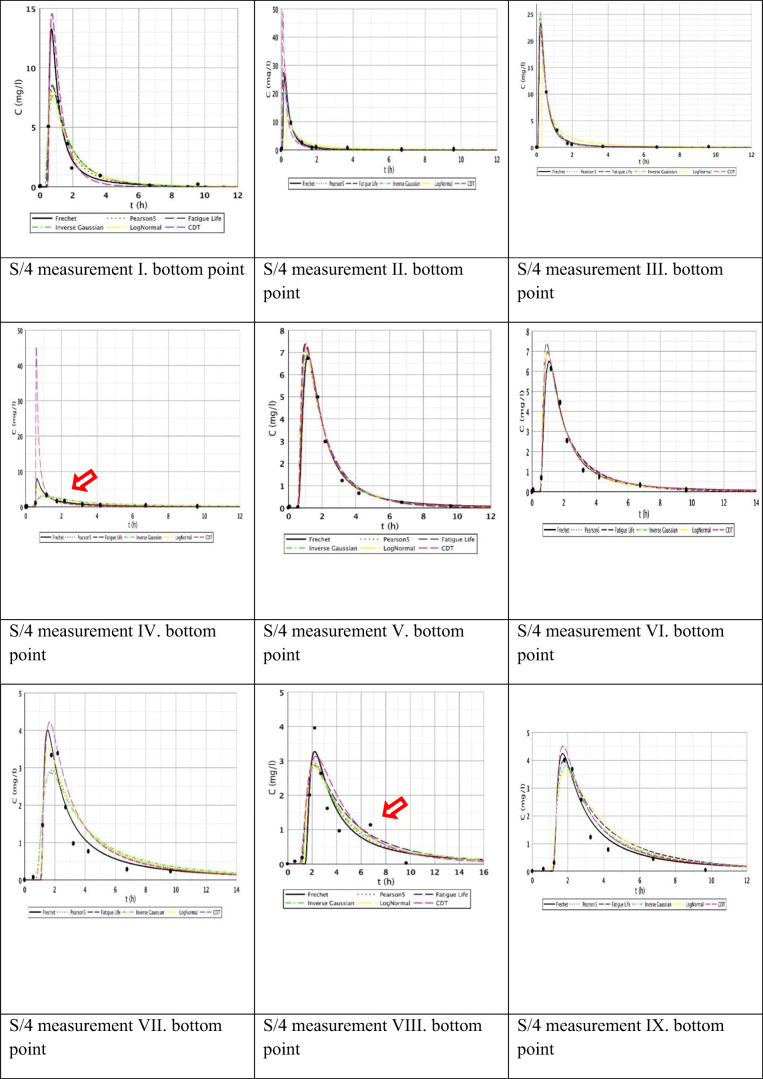
Fitting results of the five distributions and CDT model on S/4 measurement bottom points

## Appendix 1

**Table 5:** Main data of own tracer measurement

Reference number of measurement	S/1	S/2	S/3	S/4
Source	Own measurements			
Date	September 02, 2007	October 07, 2007	February 08, 2008	May 29, 2008
Type of tracer	LiCl			
Amount of tracer (g)	8–17			
Geographical location	Hódmezővásárhely, Hungary			
Source of wastewater	Milk room			
Medium	4–12 mm gravel			
Plant	Sedge			
Distribution and collection pipe	DN 125 PVC pipe perforated with 4-mm holes			
Inlet and outlet system	DN 160 PVC pipe			
Age of wetland	1 week	1.5 months	5 months	8 months
Dimensions of media body	6.85*3.7*0.6			
Evaporation at measurement (mm/d)	Negligible			
Porosity (−)	0.27	0.23	0.17	0.17
Hydraulic loading rate (m^3^/d)	29.4	31.1	16.93	14.34
Area under C-t function at 100% tracer response (h*mg/l)	6.669	6.305	11.582	13.674
Area of C-t function at 100% tracer response, corrected for evaporation (h*mg/l)	6.669	6.305	11.582	13.674
Distance between the distribution and collection pipes (m)	0.47; 2.21; 3.87	0.47; 2.21; 3.87	0.47; 2.21; 3.87	0.47; 2.21; 3.87
Porous velocity (m/d)	83.76	104.01	76.61	64.89
Parameter *b* = L/v_x_ (*d*)	0.06	0.04	0.06	0.07
Parameter *b* = L/v_x_ (*h*)	1.33	1.07	1.46	1.72

**Table 6:** Data series used for statistical research, September 02, 2007

S/1	Area under function: 66694 (h*mg/l)											
I. t	Time (h)	0.00	0.25	0.83	1.75	2.80	3.78	4.75	6.75	8.75	10.67	
	C (mg/l)	0.00	14.39	3.37	0.66	0.19	0.02	0.01	0.00	0.00	0.00	
I. b	Time (h)	0	0.25	0.8	1.8	2.8	3.8	4.8	6.8	8.8	10.7	
	C (mg/l)	0	14.4	2.4	1	0.3	0	0.1	0	0	0	
II. t	Time (h)	0	0.25	0.9	1.8	2.8	3.8	4.8	6.8	8.8	10.7	
	C (mg/l)	0	11	2.2	0.5	0.1	0	0	0	0	0	
II. b	Time (h)	0	0.25	0.9	1.8	2.8	3.8	4.8	6.8	8.8	10.7	
	C (mg/l)	0	11	3.6	0.4	0.1	0	0	0	0	0	
III. t	Time (h)	0	0.25	0.9	1.8	2.9	3.8	4.8	6.8	8.8	10.7	
	C (mg/l)	0	28.7	3.3	0.2	0	0	0	0	0	0	
III. b	Time (h)	0	0.25	0.9	1.8	2.9	3.8	4.8	6.8	8.8	10.7	
	C (mg/l)	0	28.7	4.1	0.2	0	0	0	0	0	0.04	
IV. t	Time (h)	0	0.93	1.8	3.8	4.8	6.8	8.8	11			
	C (mg/l)	0	3.5	2.7	0.3	0.1	0.1	0	0			
IV. b	Time (h)	0	0.93	1.8	2.9	3.8	4.8	6.8	8.8	11		
	C (mg/l)	0	1.86	3	1.3	0.6	0.2	0.1	0	0		
V. t	Time (h)	0	0.97	1.8	3.9	4.8	6.8	8.8	11			
	C (mg/l)	0	0.39	3.3	0.4	0.2	0.1	0	0			
V. b	Time (h)	0	0.97	1.8	3.9	4.8	6.8	8.8	11			
	C (mg/l)	0	0.3	3.6	0.6	0.2	0.1	0	0			
VI. t	Time (h)	0	0.98	1.8	3.9	4.8	6.9	8.9	11			
	C (mg/l)	0	0.71	4.2	0.7	0.2	0.1	0	0			
VI. b	Time (h)	0	0.98	1.8	3.9	4.8	6.9	8.9	11			
	C (mg/l)	0	0.64	4.3	0.9	0.4	0.1	0	0			
VII. t	Time (h)	0	0.7	1.3	1.8	2.4	2.8	3.2	4.1	5.1	7.13	9.1
	C (mg/l)	0	0.1	5.3	2.9	0.6	0.3	0.2	0.1	0.1	0.05	0.02
VII. b	Time (h)	0	0.57	1.1	1.6	2.1	2.5	3.1	3.6	4.6	9.1	
	C (mg/l)	0	0.05	2.3	3.8	1.6	0.6	0.5	0.4	0.2	0.05	
VIII. t	Time (h)	0	0.58	0.7	1.1	1.3	1.6	1.9	2.1	2.4	2.55	2.81
		3.05	3.25	3.6	4.1	4.6	5.1	7.2	9.1	11		
	C (mg/l)	0	0.05	0.1	0.3	2.6	3.6	2.9	2.2	1.2	1	0.65
		0.47	0.44	0.4	0.2	0.2	0.1	0.1	0.1	0		
VIII. b	Time (h)	0	0.58	0.7	1.1	1.3	1.6	1.9	2.1	2.4	2.57	2.8
		30.667	3.25	3.6	4.6	5.1	6.1	7.2	9.1	11		
	C (mg/l)	0	0.05	0.1	0.7	2.8	4.3	3.3	2.3	1.4	1.17	0.81
		0.52	0.58	0.4	0.3	0.2	0.3	0.1	0.1	0		
IX. t	Time (h)	0	0.73	1.4	1.9	2.4	2.8	3.3	5.1	6.2	7.18	11.15
	C (mg/l)	0	0.06	7.6	3.4	1.2	0.7	0.5	0.1	0.2	0.08	0.04
IX. b	Time (h)	0	0.6	1.1	1.6	2.1	2.6	3.1	3.6	4.6	9.15	
	C (mg/l)	0	0.05	2	6.1	2.6	1.1	0.6	0.4	0.2	0.06	

**Table 7:** Data series used for statistical research, October 10, 2007

S/2	Area under function: 6305 (h*mg/l)											
I. t	Time (h)	0	0.2	0.8	1.3	2.3	4.6	6.6	11			
	C (mg/l)	0	10.8	1	0.8	0.2	0	0	0			
I. b	Time (h)	0	0.05	0.5	1	1.9	3.6	8.6				
	C (mg/l)	0	0.05	8.1	3.6	0.6	0.1	0				
II. t	Time (h)	0	0.07	0.2	0.5	0.8	1.1	1.3	1.9	2.3	3.58	4.58
		65.667	8.58	11								
	C (mg/l)	0	0.19	7.2	5.3	2.2	0.9	0.5	0.4	0.1	0.1	0.07
		0.02	0.02	0								
II. b	Time (h)	0	0.07	0.2	0.5	0.8	1.1	1.3	1.9	2.3	3.6	4.58
		66.167	8.58									
	C (mg/l)	0	0.04	4.1	5.3	3.6	2.6	1.2	0.5	0.4	0.26	0.13
		0.02	0.01									
III. t	Time (h)	0	0.23	0.8	1.3	2.4	4.6	6.7	11			
	C (mg/l)	0	25.8	2.7	0.2	0	0	0	0			
III. b	Time (h)	0	0.08	0.5	1.1	1.9	3.6	8.6				
	C (mg/l)	0	7.73	5.7	0.5	0.2	0	0				
IV. t	Time (h)	0	0.23	0.9	1.4	1.9	2.6	3.6	4.6	6.7	10.6	
	C (mg/l)	0	2.44	3.1	0.8	0.5	0.4	0.1	0	0	0.01	
IV. b	Time (h)	0	0.1	0.5	1.1	1.6	2.1	3.1	4.1	8.6		
	C (mg/l)	0	0.8	2	2.1	1.8	0.7	0.2	0.1	0		
V. t	Time (h)	0	0.12	0.3	0.6	0.9	1.1	1.4	1.6	1.9	2.08	2.58
		3.1	3.65	4.1	4.6	6.7	8.6	11				
	C (mg/l)	0	0.05	0.1	4.7	3.5	2.1	1.2	0.8	0.5	0.45	0.28
		0.2	0.16	0.1	0.1	0	0	0				
V. b	Time (h)	0	0.12	0.3	0.6	0.9	1.1	1.4	1.6	1.9	2.08	2.6
		30.833	3.65	4.1	4.6	6.7	8.6	11				
	C (mg/l)	0	0.13	0.4	4.5	3.6	2.1	1.2	0.9	0.5	0.49	0.24
		0.33	0.16	0.1	0.1	0	0	0				
VI. t	Time (h)	0	0.27	0.9	1.4	1.9	2.6	3.7	4.7	6.7	10.6	
	C (mg/l)	0	0.62	4	1	0.3	0.2	0.1	0.1	0	0.02	
VI. b	Time (h)	0	0.13	0.6	1.1	1.6	2.1	3.1	4.1	8.7		
	C (mg/l)	0	0.04	8.1	2	0.6	0.3	0.2	0.1	0.1		
VII. t	Time (h)	0	0.27	0.9	1.4	1.9	2.4	2.8	3.7	4.7	6.7	8.66
	C (mg/l)	0	0.1	5.3	2.9	0.6	0.3	0.2	0.1	0.1	0.05	0.02
VII. b	Time (h)	0	0.13	0.6	1.1	1.6	2.1	2.6	3.1	4.1	8.67	
	C (mg/l)	0	0.05	2.3	3.8	1.6	0.6	0.5	0.4	0.2	0.05	
VIII. t	Time (h)	0	0.15	0.3	0.6	0.9	1.2	1.4	1.6	1.9	2.12	2.38
		26.167	2.82	3.1	3.7	4.1	4.7	6.7	8.7	11		
	C (mg/l)	0	0.05	0.1	0.3	2.6	3.6	2.9	2.2	1.2	1	0.65
		0.47	0.44	0.4	0.2	0.2	0.1	0.1	0.1	0		
VIII. b	Time (h)	0	0.15	0.3	0.6	0.9	1.1	1.4	1.7	1.9	2.13	2.36
		26.333	2.82	3.1	4.1	4.7	5.7	6.7	8.7	11		
	C (mg/l)	0	0.05	0.1	0.7	2.8	4.3	3.3	2.3	1.4	1.17	0.81
		0.52	0.58	0.4	0.3	0.2	0.3	0.1	0.1	0		
IX. t	Time (h)	0	0.3	0.9	1.4	2	2.4	2.8	4.7	5.7	6.75	10.71
	C (mg/l)	0	0.06	7.6	3.4	1.2	0.7	0.5	0.1	0.2	0.08	0.04
IX. b	Time (h)	0	0.17	0.7	1.2	1.7	2.2	2.6	3.2	4.2	8.72	
	C (mg/l)	0	0.05	2	6.1	2.6	1.1	0.6	0.4	0.2	0.06	

**Table 8:** Data series used for statistical research, February 08, 2008

S/3	Area under function: 11,582 (h*mg/l)									
I. t	Time (h)	0	0.05	0.7	1.2	2.3	4.5	6.5		
	C (mg/l)	0	0.01	7.6	5.3	2.6	0.9	0.6		
I. b	Time (h)	0	0.05	0.5	1	1.7	3.2	5.5		
	C (mg/l)	0	0.01	0.5	3.5	4.6	2.8	1.3		
II. t	Time (h)	0	0.05	0.7	1.2	2.3	4.5	6.5		
	C (mg/l)	0	0.01	4.2	5.6	2.1	0.8	0.5		
II. b	Time (h)	0	0.07	0.5	1	1.7	3.2	5.5		
	C (mg/l)	0	0.02	0.5	2.2	4.1	3.3	1.2		
III. t	Time (h)	0	0.07	0.7	1.3	2.3	4.5	6.5		
	C (mg/l)	0	0.01	5.1	5.9	2.1	1	0.5		
III. b	Time (h)	0	0.08	0.5	1	1.7	3.2	5.5		
	C (mg/l)	0	0.03	10	12	5.6	2.1	1		
IV.t	Time (h)	0	0.08	0.8	1.3	1.8	2.3	2.5	3.2	6.5
	C (mg/l)	0	0.01	0.7	4.3	5	1.3	3.7	2.7	0.8
IV. b	Time (h)	0	0.1	0.5	1	1.5	2	3	3.9	5.5
	C (mg/l)	0	0.04	1.6	1.1	0.5	1.9	3.3	2.3	2
V. t	Time (h)	0	0.08	0.8	1.3	1.8	2.3	2.5	3.3	6.5
	C (mg/l)	0	0.01	0.1	2	4.1	1.5	3.6	2.7	0.8
V. b	Time (h)	0	0.1	0.5	1	1.5	2	3	4	5.5
	C (mg/l)	0	0.01	0.3	0.5	3.4	4.6	3.4	2.3	1.2
VI. t	Time (h)	0	0.1	0.8	1.3	1.8	2.3	2.5	3.3	6.5
	C (mg/l)	0	0.01	0.1	2.1	4.4	1.6	41	2.8	0.8
VI. b	Time (h)	0	0.12	0.5	1	1.5	2	3	4	5.5
	C (mg/l)	0	0.01	0.1	0.9	5.4	5.7	3.9	2.8	1.5
VII. t	Time (h)	0	0.8	1.3	1.8	2.7	3.3	4.5	5.6	6.5
	C (mg/l)	0	0.03	0.2	1.6	3.6	3.3	3.1	1.9	0.8
VII. b	Time (h)	0	0.55	1.1	1.5	2	2.5	3	4	5.5
	C (mg/l)	0	0.02	0.1	0.8	1.5	3.6	2.8	2.5	1.6
VIII. t	Time (h)	0	0.82	1	1.1	2.3	2.7	3.3	4.6	6.6
	C (mg/l)	0	0.01	0	0.4	1.6	2.6	3.1	2.1	0.9
VIII. b	Time (h)	0	0.55	1	1.5	2	2.5	3	4	5.6
	C (mg/l)	0	0.02	0	0.1	0.4	2.1	1.6	2.3	1.9
IX. t	Time (h)	0	0.83	1.3	1.8	2.3	2.8	3.3	4.6	6.6
	C (mg/l)	0	0.01	0	0.3	1.2	1.9	2.6	2.1	1.1
IX. b	Time (h)	0	0.57	1.1	1.5	2	2.6	3.1	4	5.6

**Table 9:** Data series used for statistical research, May 29, 2008

S/4	Area under function: 13,674 (h*mg/l)										
I. t	Time (h)	0	0.27	0.8	1.4	2.4	4.7	8.2			
	C (mg/l)	0	2.56	3.3	0.8	0.5	0.1	0.1			
I. b	Time (h)	0	0.03	0.5	1.1	1.7	2	3.7	6.7	9.6	
	C (mg/l)	0	0.08	5.1	7.2	3.6	1.6	0.9	0.1	0.2	
II. t	Time (h)	0	0.27	0.8	1.4	2.5	4.7	8.2			
	C (mg/l)	0	4.7	2.1	0.6	0.2	0.1	0			
II. b	Time (h)	0	0.03	0.5	1.2	1.7	2	3.7	6.7	9.6	
	C (mg/l)	0	0.27	9.5	2.6	0.7	0.9	0.6	0.1	0.2	
III. t	Time (h)	0	0.28	0.8	1.5	2.5	4.7	8.2			
	C (mg/l)	0	0.58	2	1.1	0.3	0.1	0			
III. b	Time (h)	0	0.05	0.5	1.2	1.7	2	3.7	6.7	9.6	
	C (mg/l)	0	0.02	10	3.2	0.7	0.5	0.2	0	0.1	
IV. t	Time (h)	0	0.3	0.8	1.5	2	2.7	3.7	4.7	8.2	
	C (mg/l)	0	0.12	1.8	2	1.5	0.7	0.3	0.1	0.1	
IV. b	Time (h)	0	0.07	0.5	1.2	1.8	2.2	3.2	4.2	6.7	9.62
	C (mg/l)	0	0.07	1	3.3	1.6	1.6	0.7	0.4	0.4	0.09
V. t	Time (h)	0	0.32	0.8	1.5	2	2.7	3.7	4.7	8.2	
	C (mg/l)	0	0.04	0.9	4.7	3.9	1.7	0.7	0.4	0.1	
V. b	Time (h)	0	0.07	0.6	1.2	1.8	2.2	3.2	4.2	6.7	9.63
	C (mg/l)	0	0.06	0.4	6.7	5	3	1.2	0.7	0.2	0.08
VI. t	Time (h)	0	0.32	0.8	1.5	2	2.7	3.8	4.8	8.2	
	C (mg/l)	0	0.04	3.8	4.1	3.5	1.3	0.5	0.3	0.1	
VI. b	Time (h)	0	0.08	0.6	1.2	1.8	2.2	3.2	4.2	6.8	9.65
	C (mg/l)	0	0.07	0.7	6.1	4.4	2.5	1.1	0.7	0.3	0.08
VII. t	Time (h)	0	0.83	1.5	2	2.5	2.9	3.8	4.8	8.3	
	C (mg/l)	0	0.19	2.2	3	1.6	1.1	0.5	0.3	0.1	
VII. b	Time (h)	0	0.58	1.2	1.8	2.2	2.7	3.2	4.2	6.8	9.65
	C (mg/l)	0	0.07	1.5	3.3	3.4	1.9	1	0.8	0.3	0.23
VIII. t	Time (h)	0	0.87	1.5	2	2.5	3	3.8	4.8	8.3	
	C (mg/l)	0	0.05	0.9	2.8	1.8	1.7	0.9	0.5	0.2	
VIII. b	Time (h)	0	0.6	1.2	1.8	2.2	2.7	3.3	4.2	6.8	9.68
	C (mg/l)	0	0.07	0.2	2	4	2.6	1.6	1	1.1	0.02
IX. t	Time (h)	0	0.87	1.5	2	2.5	3	3.8	4.8	8.3	
	C (mg/l)	0	0.04	0.9	3.4	2.7	1.2	0.8	0.5	0.2	
IX. b	Time (h)	0	0.62	1.2	1.8	2.3	2.8	3.3	4.3	6.8	9.68
	C (mg/l)	0	0.08	0.3	4	3.7	2.6	1.2	0.8	0.5	0.04

## Appendix 2

**Table 10:** The five distribution *R*^2^ values at each point

Distribution type	S/1	S/2	S/3	S/4	Average	Rank
I. t						
Fatigue Life	0.9990483	0.9964985	0.9905653	0.8268249	0.9532342	4
Frechet	0.9954288	0.9983542	0.9804223	0.9414321	0.9789094	1
Inverse Gauss	0.9984391	0.9933385	0.9876921	0.5959973	0.8938667	5
LogNormal	0.9976400	0.9980836	0.9859843	0.8520550	0.9584407	3
Pearson	0.9959178	0.9982882	0.9813021	0.8689344	0.9611106	2
	I. b					
Fatigue Life	0.9973505	0.9997468	0.9771815	0.9362932	0.9776430	3
Frechet	0.9988952	0.9997236	0.9815055	0.9822044	0.9905822	1
Inverse Gauss	0.9982367	0.9997567	0.9657623	0.9303222	0.9735195	5
LogNormal	0.9986666	0.9998048	0.9572933	0.9459650	0.9754324	4
Pearson	0.9983096	0.9998377	0.9796206	0.9570924	0.9837151	2
II. t						
Fatigue Life	0.9999796	0.9380227	0.9881080	0.9561988	0.9705773	4
Frechet	0.9997464	0.9771032	0.9953776	0.9797063	0.9879834	2
Inverse Gauss	0.9999635	0.9647803	0.9894761	0.2362123	0.7976080	5
LogNormal	0.9999397	0.9510968	0.9963922	0.9675143	0.9787358	3
Pearson	0.9997480	0.9791859	0.9927710	0.9824567	0.9885404	1
II. b						
Fatigue Life	0.9929453	0.9859261	0.8980641	0.9934923	0.9676069	4
Frechet	0.9817928	0.9891670	0.8072736	0.9947718	0.9432513	5
Inverse Gauss	0.9901382	0.9881574	0.9841524	0.9808378	0.9858215	2
LogNormal	0.9890182	0.9851464	0.9853617	0.9370626	0.9741472	3
Pearson	0.9835861	0.9910056	0.9887829	0.9881641	0.9878847	1
III. z						
Fatigue Life	0.9866371	0.9929030	0.9863500	0.7922346	0.9395312	4
Frechet	0.9930542	0.9969969	0.9897232	0.9597313	0.9848764	1
Inverse Gauss	0.9862241	0.9930475	0.9841524	0.2528717	0.8040739	5
LogNormal	0.9896432	0.9944416	0.9853617	0.8222279	0.9479186	3
Pearson	0.9902011	0.9936564	0.9887829	0.9448932	0.9793834	2
III. b						
Fatigue Life	0.9801622	0.9810131	0.8588887	0.9974508	0.9543787	1
Frechet	0.9883591	0.9172672	0.8294235	0.9936218	0.9321679	2
Inverse Gauss	0.9791514	0.8857450	0.8554077	0.9961181	0.9291056	3
LogNormal	0.9839547	0.9818868	0.7930911	0.9505247	0.9273644	4
Pearson	0.9845854	0.8566977	0.8464156	0.9921814	0.9199700	5
IV. t						
Fatigue Life	0.9998384	0.9834074	0.7982244	0.8584564	0.9099816	3
Frechet	0.9996639	0.9880429	0.7926658	0.9526526	0.9332563	1
Inverse Gauss	0.9998450	0.8742735	0.7963954	0.6737219	0.8360589	5
LogNormal	0.9998624	0.8810614	0.7955800	0.8896934	0.8915493	4
Pearson	0.9997257	0.9896600	0.7936633	0.8858418	0.9172227	2
IV. b						
Fatigue Life	0.9993861	0.8015015	0.5531714	0.8943552	0.8121035	4
Frechet	0.9929551	0.8312120	0.5118319	0.9828929	0.8297230	2
Inverse Gauss	0.9991790	0.8142216	0.5523752	0.7452716	0.7777618	5
LogNormal	0.9993211	0.8821993	0.5575688	0.9440974	0.8457967	1
Pearson	0.9986404	0.8256657	0.5371880	0.9381725	0.8249166	3
V. t						
Fatigue Life	0.9990576	0.9600173	0.7897402	0.9316007	0.9201039	5
Frechet	0.9998970	0.9745538	0.7731059	0.9659643	0.9283802	1
Inverse Gauss	0.9880063	0.9693795	0.7860100	0.9467878	0.9225459	3
LogNormal	0.9891960	0.9557982	0.7871123	0.9518583	0.9209912	4
Pearson	0.9918065	0.9733260	0.7754781	0.9585155	0.9247815	2
V. z						
Fatigue Life	0.9990576	0.9483234	0.9582676	0.9888563	0.9736262	3
Frechet	0.9997460	0.9699938	0.9283780	0.9950464	0.9732911	4
Inverse Gauss	0.9984269	0.9638057	0.9567854	0.9906152	0.9774083	2
LogNormal	0.9994740	0.9449103	0.9537473	0.9923065	0.9726095	5
Pearson	0.9991355	0.9708838	0.9474534	0.9945290	0.9780004	1
VI. z						
Fatigue Life	0.9924795	0.9979064	0.7924666	0.9177026	0.9251388	3
Frechet	0.9906573	0.9995147	0.7745855	0.9557481	0.9301264	1
Inverse Gauss	0.9927017	0.9886506	0.7873374	0.9253559	0.9235114	4
LogNormal	0.9932749	0.7920947	0.7869983	0.9438706	0.8790596	5
Pearson	0.9939746	0.9992733	0.7774496	0.9454392	0.9290342	2
VI. b						
Fatigue Life	0.9732938	0.9984193	0.8722504	0.9812782	0.9563104	2
Frechet	0.9731266	0.9995673	0.8439338	0.9893282	0.9514890	5
Inverse Gauss	0.9738623	0.9979910	0.8702960	0.9846687	0.9567045	1
LogNormal	0.9750581	0.9980415	0.8657561	0.9859654	0.9562053	3
Pearson	0.9768433	0.9991803	0.8578517	0.9890387	0.9557285	4
VII. t						
Fatigue Life	0.9810945	0.9856496	0.9163164	0.7315068	0.9036418	3
Frechet	0.9768150	0.9762976	0.8224238	0.8235096	0.8997615	4
Inverse Gauss	0.9701262	0.9848442	0.9144720	0.6569351	0.8815944	5
LogNormal	0.9602193	0.9786853	0.9133894	0.7829279	0.9088055	2
Pearson	0.9758899	0.9823159	0.8850978	0.7943311	0.9094087	1
VII. b						
Fatigue Life	0.8047370	0.9160324	0.9379976	0.8026297	0.8653492	5
Frechet	0.9651028	0.9943321	0.9256109	0.9397179	0.9561909	1
Inverse Gauss	0.8125819	0.8784417	0.9381574	0.8392966	0.8671194	4
LogNormal	0.8480947	0.9120819	0.9372373	0.8878620	0.8963189	2
Pearson	0.8575948	0.9295408	0.9370792	0.8528600	0.8942687	3
VIII. t						
Fatigue Life	0.7719628	0.8692355	0.9856393	0.7995190	0.8565891	4
Frechet	0.9255360	0.9547435	0.9858291	0.8975049	0.9409034	1
Inverse Gauss	0.7348998	0.8819606	0.9847765	0.7332032	0.8337100	5
LogNormal	0.8136480	0.8961653	0.9862356	0.7655223	0.8653928	3
Pearson	0.7990849	0.9219356	0.9862273	0.8275554	0.8837008	2
VIII. z						
Fatigue Life	0.8453055	0.9098024	0.9083662	0.8230729	0.8716367	5
Frechet	0.9439932	0.9670718	0.8770160	0.8973011	0.9213455	1
Inverse Gauss	0.8274731	0.9161541	0.9072530	0.8364812	0.8718403	4
LogNormal	0.8903416	0.9161541	0.9056400	0.8289256	0.8852653	3
Pearson	0.8927030	0.9477708	0.8986099	0.8445824	0.8959165	2
IX. t						
Fatigue Life	0.9911774	0.9943360	0.9959835	0.6776589	0.9147889	4
Frechet	0.9989628	0.9991253	0.9944780	0.8193722	0.9529846	1
Inverse Gauss	0.9876203	0.9924518	0.9966406	0.6998849	0.9191494	3
LogNormal	0.9877172	0.9856857	0.9972227	0.6561846	0.9067026	5
Pearson	0.9928758	0.9960280	0.9983779	0.7468479	0.9335324	2
IX. b						
Fatigue Life	0.9874206	0.9933780	0.9894684	0.9145983	0.9712163	4
Frechet	0.9980923	0.9889121	0.9496186	0.9655890	0.9755530	1
Inverse Gauss	0.9808440	0.9921971	0.9892189	0.9335342	0.9739485	3
LogNormal	0.9818835	0.9904096	0.9883371	0.8713314	0.9579904	5
Pearson	0.9922996	0.9948828	0.9811671	0.9302801	0.9746574	2

## Nomenclature

CW: Constructed wetland
FSCW: Free-surface flow constructed wetland
SFCW: Subsurface flow constructed wetland
VSFCW: Subsurface flow constructed wetland with vertical flow direction
HSFCW-C: Horizontal subsurface flow constructed wetland using coarse gravel filter media
HRT: Hydraulic retention time
D [m^2^/h]: Dispersion coefficient
x [m]: Longitudinal coordinate
CDT: Convection-dispersion tank
CSTR: Continuous stirred-tank reactor
LiCl: Lithium-chloride
C [mg/l]: Concentration
L [m]: Length of seepage zone
S/1, S/2, S/3 and S/4: Reference numbers of own measurements
D-CDT: Divided convective-dispersive tank
*R*^2^: Statistical coefficient of determination
